# Visual Memory Scan Slopes: Their Changes over the First Two Seconds of Processing

**DOI:** 10.3390/vision5040053

**Published:** 2021-11-04

**Authors:** Jane Jacob, Bruno G. Breitmeyer, Melissa Treviño

**Affiliations:** 1 Department of Psychology and Behavioral Sciences, Louisiana Tech University, Ruston, LA 71272, USA; 2 Department of Psychology, University of Houston, Houston, TX 77204, USA; brunob@uh.edu (B.G.B.); melissa.trevino@nih.gov (M.T.)

**Keywords:** visual sensory memory, visual working memory, visual memory capacity, visual memory scanning

## Abstract

Using the prime–probe comparison paradigm, Jacob, Breitmeyer, and Treviño (2013) demonstrated that information processing in visual short-term memory (VSTM) proceeds through three stages: sensory visible persistence (SVP), nonvisible informational persistence (NIP), and visual working memory (VWM). To investigate the effect of increasing the memory load on these stages by using 1, 3, and 5 display items, measures of VSTM performance, including storage, storage-slopes, and scan-slopes, were obtained. Results again revealed three stages of VSTM processing, but with the NIP stage increasing in duration as memory load increased, suggesting a need, during the NIP stage, for transfer and encoding delays of information into VWM. Consistent with this, VSTM scan-slopes, in ms/item, were lowest during the first NIP stage, highest during the second NIP stage, and intermediate during the third, non-sensory VWM stage. The results also demonstrated a color-superiority effect, as all VSTM scan-slopes for color were lower than those for shape and as all VSTM storages for color are greater than those for shape, and the existence of systematic pair-wise correlations between all three measures of VSTM performance. These findings and their implications are related to other paradigms and methods used to investigate post-stimulus processing in VSTM.

## 1. Introduction

**Figure 1:**
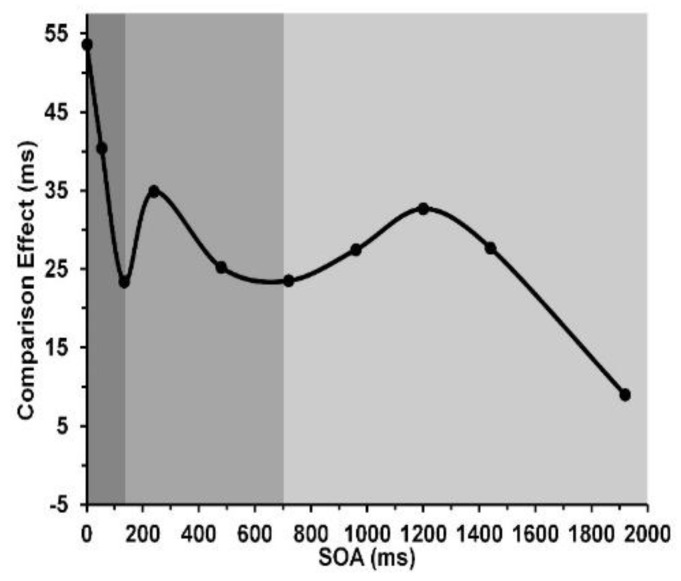
Comparison effect (RT_probe mismatch—_RT_probe match_) as a function of prime–to–probe SOA. Reprinted from Jacob et al. [14], with permission © 2013, Springer Nature.

Before a visual stimulus elicits an observer’s response, its information undergoes transformation as it is transferred through several successive stages of processing until it reaches and is stored in VWM. Traditionally the information-processing stages were conceived in terms of different visual short-term memories (VSTMs), in which information processing proceeds from the earliest, sensory memory [1] composed of sensory visible persistence (SVP) lasting about 150 ms [2], followed by a more abstract/nonvisible sensory informational persistence (NIP) that can last from about 500 to 900 ms [3,4,5,6], during which information in turn is encoded into and stored in visual working memory (VWM), whose duration can attain several seconds [7]. The information in a visual stimulus must be processed and transferred through SVP and NIP into VWM before a response can be generated. Nonetheless, one can investigate the temporal properties of all three stages [4,8,9,10,11,12,13]. Recently Jacob, Breitmeyer, and Treviño [14] developed a prime–probe comparison task that allowed tracking of these successive stages over the first two seconds of visual processing. In this study, a single prime item was presented first and was followed, at stimulus onset asynchronies (SOAs) varying from 0 to 1920 ms, by a probe. The task of the observer was to indicate as fast and accurately as possible, by pressing one of two keys, whether the probe matched or mismatched the prime. Matching trials generated faster responses than did mismatching trials. At every SOA, the comparison effect was defined as the difference between correct reaction times (RTs) obtained when the prime and probe mismatched and correct RTs obtained when the prime and probe matched. Figure 1, adapted from Jacob et al. [14], shows the comparison effects as a function of prime-to-probe SOA. As can be seen, the comparison effect fluctuated as prime-probe SOA increased: it was strongest at a prime–probe SOA of 0 ms, declined to a local minimum at an SOA of 133 ms, followed by a local maximum at 240 ms, in turn followed by a local minimum at 700 ms, and finally a local maximum at an SOA of 1200 ms. The fluctuation of the comparison effect across SOAs corresponded to the three VSTM stages of processing introduced above. In the first stage, lasting 133 ms and indicated by the dark gray area, the prime’s information accumulates (integrates) as it is processed in SVP [2]; during the second, NIP stage indicated by the mid-gray area in Figure 1 and lasting another 500 ms, the (no longer visible) information processed in NIP undergoes transfer and encoding into the third, VWM stage; and during this stage, indicated by the light gray area, information is already stored in VWM. While the entire sequential VSTM processing of a visual stimulus up to the level of VWM is accomplished in roughly 1000 ms, the storage of information in VWM can last several additional seconds.

### 1.1. Elaborations of the Temporal Properties of VSTM

One can therefore think of the Jacob et al. [8] study as a visual short-term memory (VSTM) scan study during the first two (SVP and NIP) stages of VSTM [4], combined with the procedures of Sternberg’s classic studies of VSTM scan during the VWM stage [9,10]. (We refer to what is a search through VWM as a scan of VWM, primarily because the term “search” in the recent decades has been associated with the visual-search paradigm [15,16], in which the probe precedes a study/target display that must be visually searched to decide whether the probe matched an item in the target display or mismatched all of them.) Regarding SVP, Sperling [6] found that the rate with which information in it could be scanned and transferred into later VSTM stages was about 10 ms/item. Regarding VWM, Sternberg [9,10] varied the number of items in a study display, which was followed about 2 sec later by a single probe that either matched an item or else mismatched all items in the study display. The observed task was to respond as rapidly and accurately as possible by pressing one of two buttons, indicating whether the probe matched an item or mismatched all items in the prior study display. This allowed determination of the slopes, in units of ms/item displayed, of the VWM scan. As the number of items in the study display increased, the RTs (for both the match and mismatch conditions) increased linearly, with a slope of ranging from about 40 to 50 ms/item, the exact values depending on the familiarity and complexity of the items.

In contrast to Sperling’s and Sternberg’s studies, Jacob et al.’s [14] study limited the study display to a single item, the prime stimulus, and for that reason, no scan-slopes could be determined as a function of prime-to-probe SOA. Moreover, a cooccurring VSTM storage-slope, measured in units of storage/item displayed, also could not be computed as a function of prime-to-probe SOA. The overall aim of the present study is to overcome these shortcomings by using study displays consisting of 1, 3, or 5 items, followed by a single matching or else mismatching probe. By determining the VSTM scanning and storage slopes as a function of SOA, one can obtain an estimate of the VSTMM scanning and storage efficiency at each SOA as the information processing within VSTM proceeds from the initial two, SVP and NIP, stages to the third VWM stage.

Since the processing of information through these stages requires attention, the notion of attentional episodes introduced by Wyble et al. [17] or attentional dwell times introduced by Duncan et al. [18,19] take on special importance. Attentional dwell times can last several hundreds of milliseconds [17,18,19] and are particularly important in the NIP stage during which information is transferred and encoded into VWM [11,12,13,17]. Our study importantly addresses if and how an attentional bottleneck during the NIP stage, causing attentional competition between the processing of the study display and the following probe [17], affects the efficiency of VWM storage and VWM scanning as the number of study display items increases. The efficiency was measured respectively in terms of the VSTM storage (total number of items stored), VSTM storage-slope (storage/display item), and of the VSTM scan-slope (ms/display item). By determining the storage, storage-slopes, and scan-slopes as a function of display-to-probe SOA, one can obtain an estimate of the how storage and scanning efficiencies are affected at each SOA as the information processing proceeds from the first SVP stage, to the second NIP stage, and finally to the third VWM stage.

### 1.2. Significance of Expected Findings

We already know that the SVP scan-slope is roughly 10 ms/item [8] and that the VWM scan-slope is 40–50 ms/item [9,10]. The significance of our research lies in additionally determining the scan-slope obtained in the NIP regime. Moreover, by varying the display-to-probe SOA from 0 to 1920 ms, we can also determine if and how scan-slopes change as transitions are made from SVP to NIP and thereafter from NIP to VWM. In this regard, our research is exploratory, and therefore we entertain specific expectations about the characteristics of the transitions. However, since there is attentional competition between the processing of the study display and that of the probe, we expect the NIP scan-slopes (ms/display item) to be larger than the SVP and the VWM scan-slopes.

**Figure 2:**
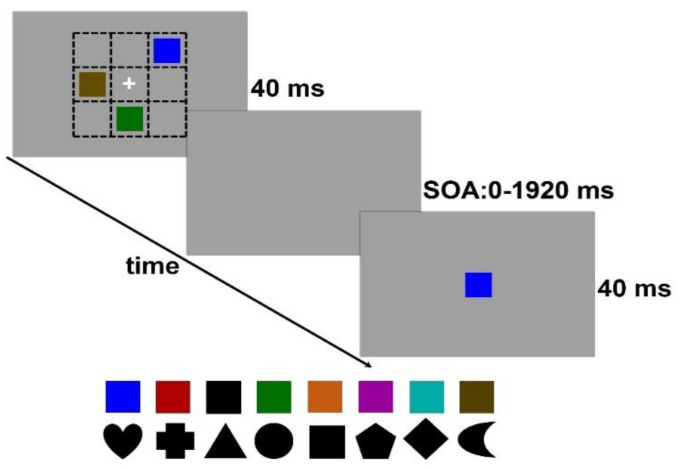
Example of color stimuli used in the VSTM scanning task. A 40–ms study display, here with a VSTM load of 3, was followed, at SOAs varying from 0 to 1920 ms, by a 40–ms probe centered at fixation. The observer was to respond as quickly and accurately as possible whether the probe (as shown) matched one or mismatched all of the items in the study display. Dashed line matrix designates the notional 3 × 3 matrix in which the central probe and the surrounding color or shape stimuli were positioned.

In the Jacob et al. [14] study, two types of stimuli, colors, and shapes were used. We also used color and shape stimuli in the present study. Relative to items differing only in a single feature dimension such as color, items differing in shape, comprised of several feature dimensions such as edge orientation, edge curvature, and number of vertices, are more complex (see Figure 2 below). Higher complexity translates into a higher informational load [20], and therefore it is reasonable to expect that we expect, based on studies of VWM finding a consistent color-superiority effect that is reflected by higher storage in VWM for color stimuli [13,20,21,22,23], that all of our three VSTM performance indicators (storage, storage-slope, and scan-slope) will be superior to those for shape stimuli.

Finally, we also expected (1) that there would be strong correlations between our three measures of VSTM performance. In particular, across display-to-probe SOAs we expected that the VSTM storages and the VSTM storage-slopes (storage/item) would correlate positively. In other words, increases and decreases of storage ought to be associated respectively with increases and decreases of storage slopes. We also expected (2) that VSTM storages and VSTM scan-slopes (ms/item) should correlate negatively. That is, higher and lower VSTM storage values ought to be associated respectively with faster and slower VSTM scanning, which is indexed by lower and higher scan-slopes. If expectations (1) and (2) hold true, it follows (3) that VSTM storage-slopes and scan-lopes should also correlate negatively.

## 2. Materials and Methods

### 2.1. Participants

University of Houston students were recruited to participate as observers in the VSTM scanning task. The sample consisted mainly of undergraduate and post-baccalaureate students. Undergraduate students received extra credit towards their psychology courses. All recruited participants reported having normal or corrected-to-normal vision. On that basis it was assumed that they also had normal color vision. Of these participants, five were females, the rest males, with ages ranging from the early to the middle twenties. Participants gave informed and voluntary consent to serve as observers. All participants were familiarized with their experimental task by verbal instruction and monitoring from the experimenter during a brief 5–10 trial practice session.

### 2.2. Apparatus

The presentation of stimuli and the recording of responses were controlled using Matlab running on a Dell Optiplex 755 computer with an Intel core 2 processor. The video-display monitor was set at 1280 × 1024 resolution and the refresh rate of the monitor was 75 Hz.

### 2.3. Stimuli and Procedure

Two types of stimuli were used for the VSTM scanning task. One type consisted of colored squares; the other, of black shapes (see bottom of Figure 2). Observers were presented a 40-ms study display of 1, 3, or 5 items. As shown in Figure 2, items could be presented at randomly chosen positions, excluding the central one, of a notional 3 × 3 matrix centered at fixation. The study display was followed at a preset SOA by a single 40-ms probe presented at fixation. The task of the observer was to determine as rapidly and accurately as possible whether or not the probe matched any item in the prior study display. The items in the study display defined the visual processing load (1, 3, or 5) across the various VSTM stages. The SOA separating the study display form the probe could take one of 10 values: 0, 40, 133, 240, 480, 720, 960, 1200, 1440, and 1920 ms. These SOAs are the same as those used by Jacob et al.’s [4] study assessing the comparison effect at various stages of post-display processing (see Figure 1). The square colored stimuli (blue, red, green, orange, violet, teal, brown) were all equiluminant at 36 cd/m^2^; the black square was set at 7 cd/m^2^. The black shapes (heart, cross, triangle, circle, square, pentagon, rhombus, and crescent) also had a luminance of 7 cd/m^2^. All stimuli, presented on a gray background (16 cd/m^2^), had a Michelson contrast of 0.39 and were roughly 1.5 × 1.5 deg at a viewing distance of 67 cm. Viewing was binocular. On half of the randomly selected trials, the central probe matched one of the items in the study display, while on the other half of the trials, the central probe mismatched all of the items in the study display. The order of VSTM load was counterbalanced across observers. Separate trial blocks were assigned to each of the shape and color stimuli in counterbalanced order. Additionally, within each of the color and shape conditions, the order of SOA-value was counterbalanced across separate blocks of trials. At SOA = 0 ms, the central probe and the surrounding study display items were presented simultaneously for 40 ms.

### 2.4. Statistical Methods

All statistical tests were performed using IBM SPSS software. Repeated measures ANOVAS were used throughout, with follow-up post-hoc *t*-tests. For both F- and t-tests, exact *p*-values were limited to 0.001; *p*-values lower than 0.001 were shown as *p* < 0.001.

## 3. Results and Discussion

The specific aims of our study were: (1) to establish how VSTM storage values (in number of items), VSTM storage-slope values (storage/memory load), and VSTM scan-slope values (ms/memory item) change over the first two seconds of post-stimulus processing. This time interval spans VSTMs beginning with NIP at the shortest study display-to-probe SOAs ranging from 0 to roughly 150 ms, followed by NIP at intermediate SOAs ranging from 150 to about 1000 ms, to and proceeding to VWM at the SOAs exceeding 1000 ms, and (2) to establish how VSTM storage values, VSTM storage-slope values, and VSTM scan-slope values relate to each other.

### 3.1. Tests of Normality and Homoscedasticity

**Figure 3:**
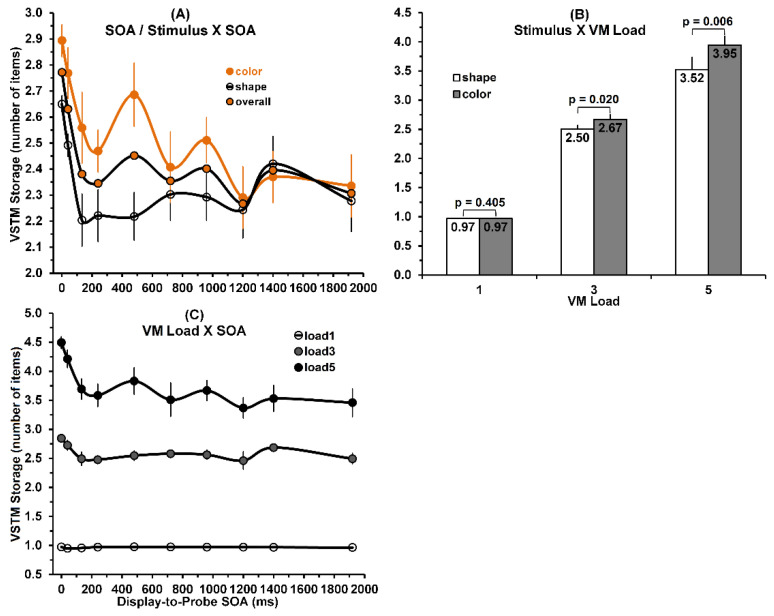
VSTM storage as a function of display–to–probe SOA. (Panel **A**): Orange, oopen, and orange–filled circles correspond respectively to color, shape, and overall storage values. The data illustrate the significant main effects of stimulus and the significant stimulus × SOA interaction. (Panel **B**): The significant stimulus × VSTM load interaction. (Panel **C**): The significant VSTM load × SOA interaction.

**Figure 4:**
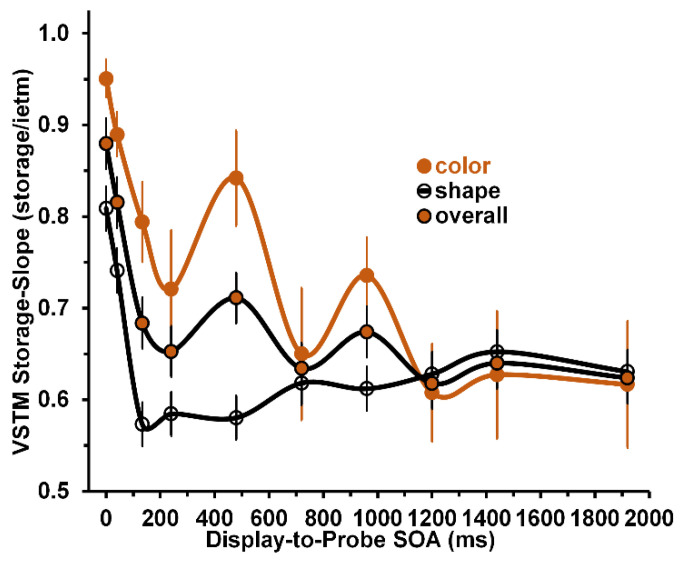
VSTM storage–slopes as a function of SOA. Orange, open, and orange–filled circles depict results obtained for color, shape, and overall results.

**Figure 5:**
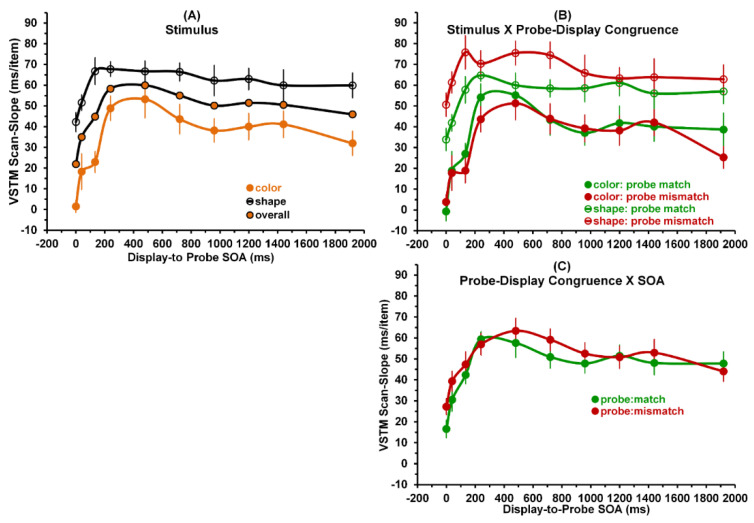
VSTM scan–slopes as a function of display–to–probe SOA. (Panel **A**): Averaged across probe–display congruences, orange, open, and orange–filled circles denote respectively color, shape, and overall results. The data show the significant main effects of stimulus and SOA. (Panel **B**): Red and green circles denote respectively probe mismatch and match conditions. Open and filled circles denote respectively shape and color stimuli. The data show the significant interaction between stimulus and probe–display congruence across SOAs. (Panel **C**): Insignificant interaction between probe–display congruence and SOA.

**Figure A1:**
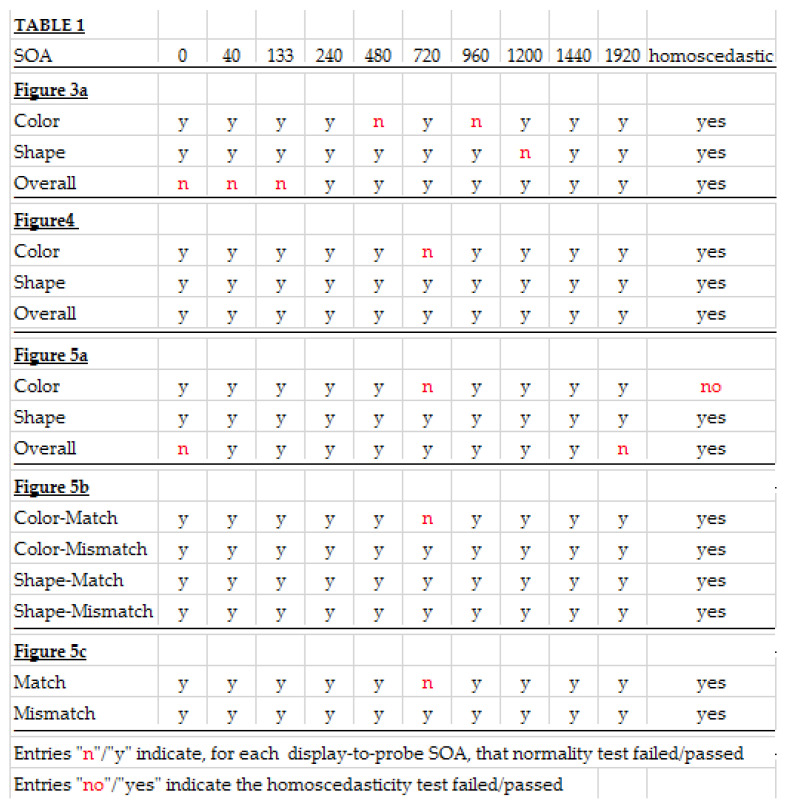
Results of normality and homoscedasticity tests for the data used in Figure 3A, Figure 4, Figure 5A–C.

We applied the Shapiro–Wilk test for normality and the Levene test for homoscedasticity to all of our data that were submitted to an ANOVA. Figure A1 (see Appendix A below) shows the results of the two tests for data shown below in Figure 3A, Figure 4 and Figure 5A–C. Of the 150 SOA entries corresponding to these five figures only 12 failed to pass the test for normality, and only 1 of 15 homoscedasticity tests failed.

Tests were not conducted for the data shown in Figure 3c, for the following reasons. Since the VMM capacity is around 4–5 items, a VWM load of 1 will produce a ceiling effect reflected in a rectangular distribution of storage values instead of a normal one. However, as the VWM load increases progressively from 3 to five the distribution has an increasing chance to approach normality. That this was indeed the case is shown by the facts that of 20 possible normality tests at each VWM load, progressively fewer—15, 6, and 4—failed the normality test as VWM load increased from 1, through 3, to 5. Moreover, since it is reasonable to expect that variability of results increased in direct proportion to increases of the VWM load from 1, through 3, to 5, a specious lack of homoscedasticity would emerge.

### 3.2. Analysis of VSTM Storage Values

To that end, we first performed a three-way (stimulus × VSTM load × SOA) repeated-measures ANOVA of storage values (During a VSTM scanning task, observers generate not only a majority of hits when they correctly respond that the probe matched an item in the prior study display, but also some false alarms when they incorrectly respond that probe matched an item in the prior study display when in fact it mismatched. Hence, one can compute hit and false-alarm rates that allow computing VSTM storage values. According to Cowan et al. [24], the appropriate formula is: storage = N(h − f)/h, where N is the memory load (1, 3, or 5), h is the hit rate, and f is the false-alarm rate). Of course, the main effect of VSTM load would turn out to be significant by design, given that the storage value obtained at each load is directly related to VSTM load. Of more interest is the fact that the main effects of stimulus [F(1,8) = 9.25; *p* = 0.016, η^2^ = 0.536; power = 0.759] and SOA [F(9,72) = 10.28; *p* < 0.001, η^2^ = 0.562; power = 1.0] were statistically significant. The effects of all three two-way interactions, stimulus × VSTM load [F(2,16) = 8.78; *p* = 0.003, η^2^ = 0.523; power = 0.935], stimulus × SOA [F(9,72) = 2.21; *p* = 0.031, η^2^ = 0.217; power = 0.858], and VSTM load × SOA [F(18,144) = 5.69; *p* < 0.001, η^2^ = 0.416; power = 1.0] also were significant, as was the effect of the three-way interaction [F(18,144) = 2.05; *p* =0.011, η^2^ = 0.204; power = 0.974].

As to the significant main effect of stimulus on VSTM storage, a comparison of the grand means for color and shape stimuli, showed that mean of 2.53 for the color stimuli was significantly higher than that of 2.33 for the shape stimuli (one-tailed t(8) = 3.04, *p* = 0.008). The main effect of stimulus, consistent with the color-superiority effect reported in previous VWM studies [13,20,21,22,23], confirms our expectation that VSTM storage values for color stimuli will be higher than those for shape stimuli.

The significant main effect of display-to-probe SOA on storage is shown by the overall results in Panel (A) of Figure 3. The value of storage decreases steeply as SOA increases from 0 to 133 ms, and decreases gradually as SOA increases beyond 133 ms. This general decrease of storage with SOA is realized in significant linear [F(1,8) = 18.58; *p* = 0.003, η^2^ = 0.699; power = 0.964], quadratic [F(1,8) = 3.73; *p* = 0.006, η^2^ = 0.632; power = 0.899], and cubic [F(1,8) = 14.72; *p* = 0.005, η^2^ = 0.648; power = 0.917] trends.

Regarding the significant stimulus × SOA interaction, Panel (A) of Figure 3 also shows that the difference in storage values between color and shape stimuli, starts out with a relatively small value of roughly 0.24 at an SOA of 0 ms, then gradually increases up to a value of roughly 0.48 at an SOA 480 ms, and then decreases as SOA increases further to 920 ms, and are all but nonexistent at still greater SOAs. Panel (B) of Figure 3, shows how the stimulus × VSTM load interaction affected the VSTM storage. When loaded with only one stimulus, the storage values for color and shape stimuli are nearly identical. However, as VSTM load takes on values of 3 and 5, the corresponding color storage values become increasingly greater than the shape storage values.

The VSTM load × SOA interaction is shown in Panel (C) of Figure 3. While the storage values decrease and fluctuate noticeably as SOA increases for a VSTM load of 5, the SOA-associated decrease and fluctuations appear to dampen, particularly for the VSTM load of 1. We believe that, by limiting VSTM loads to 3 and especially to 1, artifactual floor effects are introduced that mainly account for the VSTM load × SOA interaction (and thereby also for the significant three-way interaction).

The main effect of stimulus, as noted, is consistent with the color-superiority effect in VSTM performance, in that color stimuli yielded higher VSTM storage values than shape stimuli did. However, the stimulus × SOA interaction only partially supports the color-superiority effect, since unexpectedly and contrary to prior findings obtained in VMW studies [13,20,21,22,23], it is nonexistent at SOAs larger than 1000 ms, where the VWM regime of VSTM dominates. The significance of the nonexistent color-superiority effect at the longer SOAs is covered in the general discussion section.

### 3.3. Analysis of VSTM Storage-Slopes

We next performed a two-way (stimulus × SOA) repeated-measures ANOVA of VSTM storage-slope values. Both main effects of stimulus [F(1,8) = 10.19; *p* = 0.013, η^2^ = 0.560; power = 0.798] and of SOA [F(9,72) = 8.62; *p* < 0.001, η^2^ = 0.518; power = 1.0] were statistically significant, as was their interactive effect [F(9,72) = 2.35; *p* = 0.022, η^2^ = 0.227; power = 0.881].

The significant main and interactive effects are shown in Figure 4. As to the significant main effect of stimulus on VSTM storage-slope values, a comparison of the storage-slope values averaged across SOAs, showed that mean of 0.74 storage/item for the color stimuli was significantly higher than that of 0.64 k/item for the shape stimuli (one-tailed t(8) = 3.19, *p* = 0.006). Similar to the main effect of stimulus for VSTM storage values, the main effect of stimulus for VSTM storage-slopes, confirming the expectation that color storage-slope values will be higher than shape storage-slope values, was consistent with the color-superiority effect in VSTM performance measures. Turning to the main effect of SOA, the slopes overall (averaged across color and shape stimuli) decreased very rapidly for SOAs ranging from 0 to 133 ms, and continued to decrease more gradually across SOAs ranging from 240 to 1920 ms. This change of the decrease with SOA was reflected in significant linear and quadratic trends (both F(1,8) ≥ 9.57; *p* ≤ 0.015, η^2^ ≥ 0.545; power ≥ 0.773) comprising the main effect of SOA. The cubic trend may account for the nonmonotonic fluctuations apparent in the gradual decline of the overall slope values as SOA increases from 240 to 1920 ms. Turning to the interaction between stimulus and SOA, Figure 4 shows that, with the exception of the SOA = 720 ms, the color storage-slopes are significantly larger than the shape storage-slopes for all SOAs from 0 to 960 ms. At SOAs > 960 ms, they do not differ significantly. While the main effect of stimulus (see above) overall confirms the existence of a color-superiority effect, the significant Stimulus × SOA interaction only partly confirms its existence.

Based on the results of prior studies [13,20,21,22,23], we expected that across all SOAs, the storage-slope values for color stimuli would be larger than those for shape stimuli. However, again similar to the analysis of storage values, at SOAs larger than 1000 ms, the obtained storage-slopes for colors were not larger than those for shapes; if anything, they were smaller. This discrepancy between expectation and outcome also is covered in the General Discussion section.

### 3.4. Analysis of VSTM Scan-Slopes

We next conducted a three-way (stimulus × probe-display congruence × SOA) repeated-measures ANOVA on only the VSTM scanning results. Of the three main effects, only stimulus [F(1.8) = 68.57; *p* < 0.001, η^2^ = 0.896; power = 1.0] and SOA [F(9,72) = 9.11; *p* < 0.001, η^2^ = 0.532; power = 1.0] were statistically significant. Of all interactive effects, only the two-way interaction between stimulus and probe-display congruence was significant [F(1.8) = 24.38; *p* = 0.001, η^2^ = 0.753; power = 0.99]. These results are depicted in Figure 5.

As to the significant main effect of stimulus on VSTM scan-slope values, a comparison of the grand means for color and shape stimuli, showed that mean of 34.00 ms/item for the color stimuli was significantly lower than that of 60.66 ms/item for the shape stimuli (one-tailed t(8) = 8.61, *p* < 0.001). Moreover, inspection of Panel (A) of Figure 5 shows that, averaged across probe-display congruence conditions, VSTM scan-slopes for color were consistently and noticeably lower across all SOAs than VSTM scan-slopes for shape. As with the significant main effects of stimulus for VSTM storage and WSTM storage-slope values, the main effect of stimulus for VSTM scan-slopes, confirming our expectation that VSTM scan-slope values or color stimuli will be lower than those for shape stimuli, was consistent with the general color-superiority effect in VSTM performance measures. Moreover, as to the main effect of SOA, the overall results show that the slopes increase rapidly as SOA increases from 0 to about 400 ms, and declines gradually as SOA increases further to 1920 ms. A combination of significant linear, quadratic trends, and cubic trends [all F(1,8) ≥ 8.78; *p* ≤ 0.018, η^2^ ≥ 0.523; power ≥ 0.738].

Panel (B) of Figure 5 illustrates the significant interaction between stimulus and probe-display congruence. The results for shape stimuli differ from those for color stimuli. For shape stimuli, the VSTM scan-slopes for probe matches were systematically lower across display-to-probe SOAs than the VSTM scan-slopes for probe mismatches. In contrast, for color stimuli, no consistent differences exist across SOAs between VSTM scan-slopes probe matches VSTM scan-slopes for mismatches. Panel (C) shows the insignificant interaction between probe-display congruence and SOA. Together, Panels (B) and (C) only partially support our expectation that, across all display-to-probe SOAs, VSTM scan-slopes for the probe-match and probe-mismatch conditions should be nearly identical. Note that results in Panel (C) indicate that there is no systematic difference across SOAs between the VSTM scan-slopes of the probe-match and probe-mismatch conditions, and thus are consistent with Sternberg’s [9,10] previous findings. Yet Panel (B) shows that, the probe-match and probe-mismatch slopes were nearly identical for the color stimuli, while for shape stimuli probe-match slopes were systematically lower than probe mismatch slopes across all SOAs, and thus not consistent these previous findings.

### 3.5. Relations between VSTM Storages, Storage-Slopes, and Scan-Slopes

**Figure 6:**
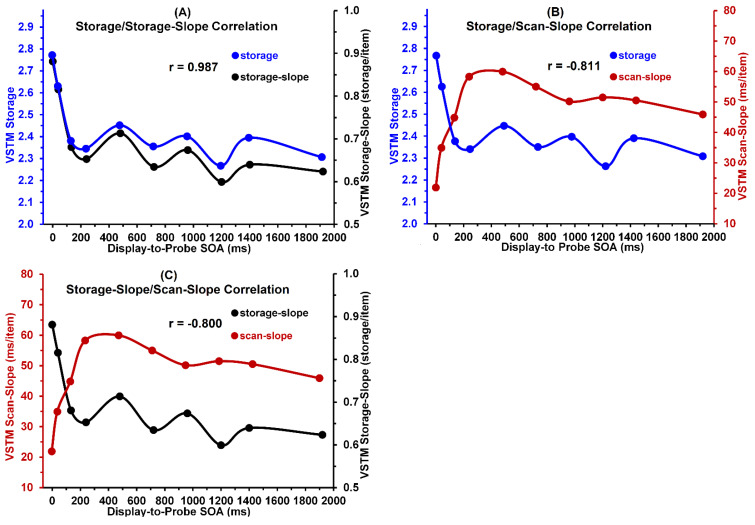
(Panel **A**): Correlations between VSTM storage and storage–slopes. (Panel **B**): Correlations between VSTM storage and scan–slopes. (Panel **C**): Correlations between VSTM scan–slopes and storage–slopes. VSTM storage, storage–slopes, and scan–slopes were averaged across color and shape stimuli.

To assess the relations, we first computed averages of the storage, storage-slope, and scan-slope values obtained with the color and shape stimuli. The Pearson r correlations shown in Figure 6 are based on use of these averaged values. We expected that, across display-to-probe SOAs, VSTM storages correlate positively with VSTM storage-slopes. Figure 6A shows that this expectation is robustly confirmed by a robust correlation of 0.987, *p* < 0.001.

We also expected that VSTM storage should correlate negatively with VSTM scan-slope. As is evident from inspection of Figure 6B, this expectation also was confirmed. The correlation between VSTM storage and VSTM scan-slope was a strong −0.811, *p* = 0.004. Additionally, as expected, Figure 6C shows that the correlation between VSTM scan-slope and VSTM storage-slope was an equally strong −0.800, *p* = 0.005.

## 4. General Discussion

Our main aim was to investigate how VSTM storage, VSTM storage-slopes, and VSTM scan-slopes varied as function of (a) stimulus type (color, shape), (b) the probe’s congruency relation to the VSTM study display by either matching some item or else mismatching all items in the study display, and (c) the SOA separating the onset of the VSTM study display from the onset of the following VSTM probe. A secondary aim was to investigate if and how VSTM storages, VSTM storage-slopes, and VSTM scan-slopes relate to each other. To accomplish these goals, we replicated and expanded Jacob et al.’s [14] single-item methodology by using VSTM study displays that contained 1, 3, or 5 items.

### 4.1. Relation of Present Findings to Those of Prior Studies

In the last two decades, a majority of prior studies of visual memory have focused on clarifying the properties of VWM. In these studies, the SOA separating the onset of the VSTM study display from the onset of the VSTM probe was typically somewhere around 1000 ms or longer. While in our study some findings at these SOAs yielded results that are consistent with those of prior VWM studies, others are not. Prior studies of VWM [13,20,21,22,23] had generally found a color-superiority effect, manifested by greater VWM storage for (simple) color than for (complex) shape stimuli. The grand mean storage value of 2.53 for color stimuli was significantly larger than the storage value of 2.33 for shape stimuli. However, evident in Panel (A) of Figure 3 is that the obtained VSTM storages (averaged over VSTM load) confirm the color-superiority effect at display-to-probe SOAs ranging from 0 to 960 ms, but not at longer SOAs. In contrast, at the display-to-probe SOAs higher than 1000 ms, VWM storage values are all but identical for color and shape stimuli.

In line with these storage results, the grand mean storage-slope value of 0.74/item obtained with color stimuli, was significantly larger than the grand mean storage-slope value of 0.64 obtained with shape stimuli. But again, Panel (A) of Figure 4 shows that the obtained VSTM storage-slopes confirm this color superiority effect only at SOAs ranging from 0 to 960 ms. In contrast, at the display to probe SOAs higher than 1000 ms, VWM storage-slopes shown in Figure 4—whose values, in units of storage/item, were assumed to be directly related to VWM storage—are all but identical for color and shape stimuli. In fact, if anything, the color storage-slopes were slightly less than the shape storage-slopes. Since (a) at SOAs longer than 1000 ms the relevant VSTM processing occurs in VWM and (b) color-superiority effect has been reported in several VWM studies [13,20,21,22,23], our contrary results obtained at these longer SOAs require explanation. After inspecting the methodology of the prior studies, we noted two important differences between their and our methodologies. First, in the prior studies the VWM study display was presented for 150 ms and longer, and after a delay it was followed by a probe display that also lasted several hundred ms. In contrast, in our study both the VSTM study display and the probe were presented for a brief 40 ms. Second, the probe display in our study consisted of a single, centrally viewed item, whereas in the prior studies the probe display could consist of several items, possibly increasing the difficulty of comparing the probe to the study display. Both of these methodological differences could have contributed to the fact that our storage and storage-slope results are inconsistent with the VWM color-superiority effect.

Sperling et al. [25] used a rapidly presented sequence of briefly flashed 3 × 3, multi-letter displays. Embedded within this sequence was a brief multi-element display, which at a randomly chosen location of the array, contained a number instead of a letter. The task of the observer was to detect the array-location of the number. Results showed that in this sequential visual-scanning task, observers were able to scan the displays very rapidly, at a rate of about 10 ms/array item. This scan-slope value, concurring with Sperling’s earlier study of VSTM [8], was significantly shorter than the average scan-slope of roughly 42 ms/item obtained in Sternberg’s VWM scanning studies [10]. We suggest that the most likely temporal property of VSTM that determined the scanning rate in Sperling et al.’s [25] study was the early SVP stage of VSTM. This interpretation of Sperling et al.’s [25] results was generally consistent with our findings. As shown by the overall results in Panel (A) of Figure 5, the VW scanning slope at the SOA of 0 ms, where observers are relying on processing in the early SVP stage of VSTM, was a relatively low 22 ms/item. In contrast, at the SOA of 1920 ms, where observers are relying on processing in the late, non-sensory VWM stage of VSTM, the VSTM scan-slope was a relatively high 46 ms/item.

Several investigators [11,12,13] have shown that encoding/consolidation of color in VWM is faster than that of shape. Our results confirmed this conclusion. As Figure 5A demonstrates, this color-superiority, indicated by smaller scan-slopes, clearly holds for all display-to-probe SOAs. These SAOs of course include the SOAs ranging from 240 to 960 ms that characterize the NIP stage of processing, during which information was encoded into VWM.

### 4.2. A Bigger Picture: The Nonmonotonic, Nonlinear Function Relating WSTM Scan-Slopes to SOA

Slopes, in units of ms/item, provided an inverse measure of the efficiency of VSTM processing and a direct measure of the strength of VSTM encoding and storage, in units of storage/item. As shown in Panel (A) of Figure 3, for overall results (averaged across color and shape stimuli), the VSTM-scan-slopes increased steeply as display-to-probe SOA increased from 0 to 480 ms and decreased gradually, with moderate fluctuation, at progressively longer SOAs. We believe that we can propose a reasonable account for the overall curvilinear trend. We adopted and adapted the key notions of Wyble et al.’s [17] attentional episodes or of Duncan and collaborators’ [18,19] attentional dwell times. Estimates of both are revealed experimentally when successively presented stimuli compete for attentional processing. Duncan et al. [18,19] reported an estimate of the attentional dwell time of about 450 ms. This also corresponds to roughly the duration of the attentional episode obtained in attentional-blink (AB) studies [26,27].

Results of several additional AB studies [28,29,30,31] indicate that the impairment of the encoding of the second of two stimuli into VWM, is as much a matter of a delay of the encoding as it is a degradation of the informational load of the second stimulus. Moreover, the attentional demand placed on the processing of the WM study display should increase progressively as VM load increases from 1, through 3, to 5. We propose that this increasing demand is realized in the progressive increase of the duration of the attentional episode or dwell time devoted to the items in the study display. What are the consequences of this for explaining the results shown in Panel (A) of Figure 5?

For answers, we refered to the overall results shown in Panel (A) of Figure 5. At a display-to-probe SOA of 0 m, an observer’s VSTM performance relied on the earliest SVP stage of VSTM processing of the study display and probe; whereas at an SOA of 1920 ms, the observer relies on the later, non-sensory VWM stage of processing the two stimuli. Clearly, at an SOA of 0 ms both the display items and the probe registered simultaneously in SVP and were therefore read-out very rapidly [8] and transferred less rapidly through NIP [11,12,13] into VWM. Here, one need not switch from one attentional window for the display to another for to the probe. Hence, a fairly low scan-slope of 22 ms/display item was obtained. Equally clearly, at a display-to-probe SOA of 1920 ms, the study display and the probe were processed in separate, noncompeting attentional windows. Here, an intermediate scan-slope of 46 ms/display item was obtained, which is very close to the average VWM scan-slope of 42 ms/display item reported by Sternberg [9,10]. It is only at SOA values ranging from about 240 to 920 ms, i.e., in the NIP regime of stimulus processing during which information was encoded into VWM, that one would expect the significant competition between attentive processing of the display items and the aftercoming probe [17,28,29,30,31]. Taking the SOA at which peak competition occurs to also be to be the one causing the largest scan-slope, inspection of overall results shown in Panel (A) of Figure 5 shows it to be roughly 480 ms, which could be taken as the average duration of the attentional episode generated by the study displays containing 1, 3, and 5 items.

Generalizing this theoretical account from the overall results shown in Panel (A) of Figure 5, to the separate color and shape results, we can also explain the systematic differences across SOAs between the high VSTM scan-slopes for shape stimuli as compared to the low VSTM scan-slopes for color stimuli. The difference, a manifestation of the color-superiority effect in VSTM processing, is due to the fact that the attentional demands placed on the relatively simple color stimuli (low informational load) are lower than on the demands placed on the more complex shape stimuli (high informational load). This converts into longer delays of encoding the probe into VWM when shape stimuli are used compared to when color stimuli are used, which in turn manifests in higher VSTM scan-slopes for shapes than for colors. Moreover, since our shape stimuli are more complex than for color stimuli, one would expect the average attentional window for shape stimuli to be longer than that for color stimuli.

Confirmation of the proposals that the attentional window increases as the number of display items increases or as the complexity/informational load of a single display item increases await the results of future experiments designed specifically to address if and how display stimulus complexity or number affect attentional windows.

### 4.3. Ecological, Real-Life Relevance of Our Findings

In the present study, the static displays contained two types of stimulus features, its surface color and its contoured shape. We believe that our results will generalize to other stimulus features such as achromatic surface brightness (gray level) and surface texture. In real life, however, we are often faced with objects that move. For instance, in athletic sports such as lacrosse or basketball, a spectator is able to visually track the motion path of a ball, and a hunter is able to visually track the motion path of, say, a flying grouse, and accordingly move their shotgun smoothly in the direction of the grouse. This raises the question as to whether the present findings generalize relevantly to another stimulus feature, viz., its motion. Our findings of a nonmonotonic, nonlinear function relating VSTM scan-slopes to SOA indeed show a clear resemblance to findings obtained by Martinez–Garcia et al. [32,33] in their human factors research on human visually guided tracking.

In Martinez–Garcia et al.’s [32] experiment, an observer was placed before viewing monitor across which a target dot starts to move along the left-right axis. A small circular stimulus whose spatial position could be controlled by the observer via a servo-device (joystick or steering wheel) under his/her control also was present on the monitor. After the target began to move, the observer’s task was to move the circular cursor via the servo-device so that its spatial displacements match as close as possible those of the moving target. The onset of the cursor was either simultaneous with that of the target or it was be delayed in 50-ms steps from 50 ms to 1000 ms relative to the onset of the target’s motion. If the observer’s visually guided tracking ability is perfect, the spatial position displacements of the circle will match exactly the position displacements of the target; and the observer’s tracking gain, which measures the ratio of the cursor’s positional displacements to that of the moving target’s positional displacements, is 1.0. Such perfection is elusive, since the positional displacements of the cursor generally lag behind those of the moving target, thus yielding a gain less than 1.0. To accomplish the tracking task, the observer must rely on procedural, visuomotor memory when the tracking task is delayed relative to the onset of the target’s motion. Using tracking gain to index visuomotor memory performance, Martinez–Garcia et al. [32] were able assess how variations of the visuomotor memory performance depends on visual tracking delay.

**Figure 7:**
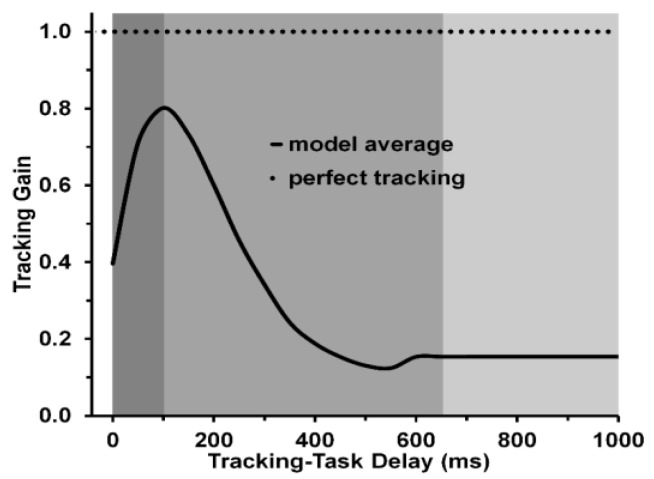
Tracking gain in a visually guided tracking task (moving a cursor on a display monitor via a servo–device) as a function of the tracking–delay after the onset of a moving target stimulus that is to be tracked by the cursor. Perfect tracking would move the cursor along the spatial locations matching that of the moving target. Gain values lower than 1.0 indicate that the spatial locations of the cursor lag behind the locations of the target. The model–response function was based on the averaged output of the model averaged over 5 values of one of its several parameters. The model–response function shows (i) that tracking is optimal, with a gain of 0.8, at a tracking–delay time of 100 ms and (ii) that the nonlinear fluctuations of the function begin to stabilize at a value of 0.15 between tracking–delay times of 650 and 700 ms (Adapted from Martinez–Garcia et al. [32], with permission).

Their results showed that the tracking-gain function indeed varied systematically as the tracking delay increased. A typical result, adapted from their Figure 7b, is shown in our Figure 7. As the tracking-onset delay increased from 0 to 1000 ms, the resulting gain function showed an initial rapid rise, attaining a maximum value of 0.8 at a delay of 150 ms, followed by a somewhat less rapid decline as delays further increased, until the gain stabilized at a value of 0.15 for delays ranging from roughly 650 to 1000 ms. What is striking about these results, here partitioned into three phases, is that the durations of the phases closely resemble the durations of the three phases indicated by Jacob et al.’s [14] findings of comparison effects shown in Figure 1. In Jacob et al.’s [14] study, the estimated first SVP stage ended at 133 ms and the second NIP stage ended at 700 ms, followed by the VWM stage. By comparison, in the Martinez et al. [32] study, the estimated first SVP stage ended at 150 ms and the second NIP stage ended at roughly 650 ms, followed by the VWM stage. Since, as noted in the Introduction, visual sensory memory consists of SVP and NIP, we can conclude from both studies that the duration of visual sensory memories for color, shape, and motion are roughly equal.

## Data Availability

The data that support the findings of this study are available from the author upon request.

## References

[1] Atkinson R.C., Shiffrin R.M., Spence K.W., Spence J.T. (1968). Human memory: A proposed system and its control processes. The Psychology of Learning and Motivation: Advances in Research and Theory.

[2] Di Lollo V. (1977). Temporal characteristics of iconic memory. Nature.

[3] Coltheart M. (1980). Iconic memory and visible persistence. Percept. Psychophys..

[4] Sperling G. (1960). The information available in a brief visual presentation. Psychol. Monogr..

[5] Dixon P., Di Lollo V. (1991). Effects of display luminance, stimulus type, and probe duration on visible and schematic persistence. Canad. J. Psychol..

[6] Irwin D.E., Yeomans J.M. (1986). Sensory registration and informational persistence. J. Exp. Psychol. Hum. Percept. Perform..

[7] Cowan N. (2010). The magical mystery four: How is working memory capacity limited, and why?. Cur. Dir. Psychol. Sci..

[8] Sperling G. (1963). A model for visual memory tasks. Hum. Fact..

[9] Sternberg S. (1966). High-speed scanning in human memory. Science.

[10] Sternberg S. (1969). Memory-scanning: Mental processes revealed by reaction time experiments. Amer. Scient..

[11] Vogel E.K., Woodman G.F., Luck S.J. (2006). The time course of consolidation in visual working memory. J. Exp. Psychol. Hum. Percept. Perform..

[12] Woodman G.F., Vogel E.K. (2005). Fractionating working memory: Encoding and maintenance are independent processes. Psychol. Sci..

[13] Woodman G.F., Vogel E.K. (2008). Selective storage and maintenance of an object’s features in visual working memory. Psychon. Bull. Rev..

[14] Jacob J., Breitmeyer B.G., Treviño M. (2013). Tracking the first two seconds: Three stages of visual information processing?. Psychon. Bull. Rev..

[15] Treisman A., Gormican S. (1988). Feature analysis in early vision: Evidence from search asymmetries. Psychol. Rev..

[16] Wolfe J.M. (1994). Guided Search 2.0: A revised model of visual search. Psychon. Bull. Rev..

[17] Wyble B., Potter M.C., Bowman H., Nieuwenstein M. (2011). Attentional episodes in visual perception. J. Exp. Psychol. Gen..

[18] Duncan J., Ward R., Shapiro K. (1994). Direct measurement of attentional dwell time in human vision. Nature.

[19] Ward R., Duncan J., Shapiro K. (1996). The slow time-course of visual attention. Cogn. Psychol..

[20] Alvarez G.A., Cavanagh P. (2004). The capacity of visual short-term memory is set both by visual information load and by number of objects. Psychol. Sci..

[21] Awh E., Barton B., Vogel E.K. (2007). Visual working memory represents a fixed number of items regardless of complexity. Psychol. Sci..

[22] Eng H.Y., Chen D., Jiang Y. (2005). Visual working memory for simple and complex visual stimuli. Psychon. Bull. Rev..

[23] Wheeler M.E., Treisman A.M. (2002). Binding in short-term visual memory. J. Exp. Psychol. Gen..

[24] Cowan N., Blume C.L., Saults J.S. (2013). Attention to attributes and objects in working memory. J. Exp. Psychol. Learn. Mem. Cogn..

[25] Sperling G., Budiansky J., Spivak J.G., Johnson M.C. (1971). Extremely rapid visual search: The maximum rate of scanning letters for the presence of a numeral. Science.

[26] Raymond J.E., Shapiro K.L., Arnell K.M. (1992). Temporary suppression of visual processing in an RSVP task: An attentional blink?. J. Exp. Psychol. Hum. Percept. Perform..

[27] Shapiro K.L., Raymond J.E., Arnell K.M. (1994). Attention to visual pattern information produces the attentional blink in RSVP. J. Exp. Psychol. Hum. Percept. Perform..

[28] Craston P., Wyble B., Chennu S., Bowman H. (2009). The attentional blink reveals serial working memory encoding: Evidence from virtual and human event-related potentials. J. Cogn. Neurosci..

[29] Nieuwenstein M.R., Chun M.M., van der Lubbe R.H.J., Hooge I.T.C. (2005). Delayed attentional engagement in the attentional blink. J. Exp. Psychol. Hum. Percept. Perform.

[30] Vogel E.K., Luck S.J. (2002). Delayed working memory consolidation during the attentional blink. Psychon. Bull. Rev..

[31] Vul E., Nieuwenstein M., Kanwisher N. (2008). Temporal selection is suppressed, delayed, and diffused during the attentional blink. Psychol. Sci..

[32] Martínez-García M., Zhang Y., Gordon T. (2021). Memory pattern identification for feedback tracking control in human–machine systems. Hum. Fact..

[33] Martínez-García M., Gordon T., Shu L. (2017). Extended crossover model for human-control of fractional order plants. IEEE Access.

